# Normal Range Albuminuria and Metabolic Syndrome in South Korea: The 2011–2012 Korean National Health and Nutrition Examination Survey

**DOI:** 10.1371/journal.pone.0125615

**Published:** 2015-05-15

**Authors:** Si-Young Park, Yong-Kyu Park, Kyung-Hwan Cho, Hee-Jeong Choi, Jee-Hye Han, Kyung-Do Han, Byung-Duck Han, Yeo-Joon Yoon, Yang-Hyun Kim

**Affiliations:** 1 Department of Family Medicine, Eulji University College of Medicine, Daejeon, South Korea; 2 Department of Medical Statistics, Catholic University College of Medicine, Seoul, South Korea; 3 Department of Family Medicine, Korea University College of Medicine, Seoul, South Korea; University of Glasgow, UNITED KINGDOM

## Abstract

**Background:**

It is well-known that there is a close relationship between metabolic syndrome (MetS) and microalbuminuria. However, some recent studies have found that even normal range albuminuria was associated with MetS and cardiometabolic risk factors. The purpose of this study is to analyze the relationship between MetS and normal range albuminuria and to calculate the cutoff value for albuminuria that correlates with MetS in the representative fraction of Korean population.

**Methods:**

Data were obtained from the 2011–2012 Korea National Health and Nutrition Examination Survey and included 9,650 subjects aged ≥19 years. We measured metabolic parameters: fasting blood glucose, waist circumference, blood pressure, and lipids, and albumin-to-creatinine ratio (ACR). The optimal ACR cutoff points for MetS were examined by the receiver operating characteristic curve. Multivariate logistic regression was used to obtain the prevalence of MetS and its components according to the ACR levels.

**Results:**

The first cutoff value of ACR were 4.8 mg/g for subjects with ≥3 components of MetS. There was a graded association between ACR and prevalence of MetS and its components. If ACR was <4 mg/g, there was no significant increase in the prevalence of MetS or its components. From the ACR level of 4–5 mg/g, the prevalence of MetS significantly increased after adjusting for age, sex, body mass index, smoking, alcohol intake, exercise, and medications for diabetes mellitus and hypertension (odds ratio; 95% confidence intervals = 1.416; 1.041–1.926).

**Conclusions:**

Albuminuria within the normal range (around 5 mg/g) was associated with prevalence of MetS in the Korean population.

## Introduction

Microalbuminuria, which is generally defined as a urinary albumin-to-creatinine ratio (ACR) of 30–300 mg/g [1], was associated with an elevated risk for cardiovascular disease and death in general population as well as in patients with diabetes mellitus (DM) and hypertension (HTN) [2–5]. Metabolic syndrome (MetS) is the most useful and widely accepted description of the cluster of metabolic abnormalities related to cardiovascular risk factors [6]. Several studies have reported a close relationship between MetS and microalbuminuria [7–10]. However, some recent studies have found that even normal range albuminuria was associated with MetS and cardiometabolic risk factors [11–15]. Jassen et al. found that low-grade albuminuria, which is far below the microalbuminuric range, was associated with cardiovascular risk factors in a sample of 40,619 Caucasians [13]. Vyssoulis et al. found that low-grade albuminuria was significantly associated with the prevalence of MetS in 6,650 hypertensive patients [11]. Two Asian studies also showed low-grade albuminuria to be positively associated with MetS in middle aged/elderly Chinese and middle-aged Korean men [14, 15]. In addition, other studies have showed that even normal range, very low-grade albuminuria is associated with elevated incidence of DM, HTN, central obesity [16–18], and cardiovascular disease [19–21]. Several studies already examined Korean population, but these studies only included middle-aged men or subjects undergoing a medical check-up. In addition, there was no study that investigated the cutoff value of albuminuria predictive of MetS in the representative fraction of Korean population.

Therefore, we investigated the relationship between normal range albuminuria and MetS and calculated a novel cutoff value for albuminuria predictive of MetS in the Korean whole population.

## Materials and Methods

### Subjects

We analyzed the data from the 2011–2012 Korea National Health and Nutrition Examination Survey (KNHANES). KNHANES has been performed since 1998 by the Division of Chronic Disease Surveillance at the Korean Center for Disease Control and Prevention and is a nationwide survey assessing national health and nutritional states. The survey consists of 3 parts: a health interview survey, a health examination survey, and a nutrition survey [22]. A total of 12,859 subjects aged ≥19 years were included in the survey. We excluded subjects who had malignancy, liver cirrhosis, chronic hepatitis B or C, thyroid disease, renal disease, or pulmonary or extrapulmonary tuberculosis, and those who were pregnant. We also excluded participants with missing data or who had not fasted for the required 8 hours before blood sample collection. A total of 9,650 subjects were included in the present study. All subjects provided written informed consent, and the study protocol was approved by the institutional review board of the Korean Center for Disease Control and Prevention.

### Anthropometric and biochemical measurements

The heights (cm) and weights (kg) of the subjects were measured to the nearest 0.1 cm and 0.1 kg, respectively, with light clothing on and shoes removed. Waist circumference (WC) was measured to the nearest 0.1 cm on a horizontal plane at the midpoint level between the iliac crest and the costal margin at the end of normal expiration. Body mass index (BMI) was calculated by dividing the weight (kg) by the square of height (m^2^). Blood pressure (BP) was measured thrice using a mercury sphygmomanometer (Baumanometer; W. A. Baum Co., Inc., Copiague, NY, USA). Each subject was seated and rested for at least 5 minutes before BP was measured. The final resting BP value was calculated by taking the average of the second and third measurements. We used this calculated average BP value for statistical analysis. Blood samples were obtained after at least 8 hours of fasting and were analyzed for serum levels of fasting blood glucose (FBG), total cholesterol (TC), high-density lipoprotein-cholesterol (HDL-C), triglycerides (TG), and low-density lipoprotein-cholesterol (LDL-C). We used the first morning urine samples of subjects. We checked urine and serum creatinine levels and the urine albumin level by kinetic colorimetry and turbidimetric assays by using the Hitachi Automatic Analyzer 7600. Albuminuria was calculated by a urine albumin-to creatinine ratio. We also calculated the estimated glomerular filtration rate (eGFR; mL/min/1.73 m^2^) using the following equation from the Chronic Kidney Disease Epidemiology Collaboration (CKD-EPI): eGFR (mL/min/1.73 m^2^) = 141 × min(serum creatinine/κ, 1)^α^ × max(serum creatinine/κ, 1)^−1.209^ × 0.993^age^ × 1.018 [if female] × 1.159 [if African American], where κ is 0.7 for women and 0.9 for men, α is -0.411 for men and -0.329 for women, min indicates minimum serum creatinine/κ or 1, and max indicates maximum serum creatinine/κ or 1 [23].

### Lifestyle variables

Alcohol consumption, smoking status, and physical activity were measured using the self-report questionnaire. Alcohol use was determined based on the amount of alcohol consumed per day during the 1-month period before the baseline interview; heavy drinkers were defined as those with alcohol consumption of ≥30 g/day. Current smokers were defined as those who were smoking currently and had smoked ≥100 cigarettes over their lifetime. The amount of physical activity performed was assessed using the International Physical Activity Questionnaire short form modified for the Korean population [24]. Subjects were divided into 2 groups: exercise and non-exercise. Regular physical exercise was defined as moderate intensity exercise for more than 5 times per week for more than 30 minutes or vigorous intensity exercise for more than 3 times per week for more than 20 minutes.

### Definition of MetS

MetS was defined according to the American Heart Association/National Heart, Lung, and Blood Institute Scientific Statement (AHA/NHLBI) criteria for Asians [25]. Participants with 3 or more of the following 5 criteria were defined as having MetS: high WC (≥90 cm for men, ≥80 cm for women), high TG (≥ 150 mg/dL or use of anti-dyslipidemic medication), low HDL-C (men < 40 mg/dL, and women < 50 mg/dL or use of anti-dyslipidemic medication), high BP (≥130/85 mmHg or use of antihypertensive drug treatment), and high BG (≥100 mg/dL or on use of drug treatment for elevated glucose levels).

### Statistical analyses

Data are presented as either means ± standard errors (SEs) for continuous variables or as percentage (SE) for categorical variables. The chi-squared test was used for categorical variables and t-test was used for continuous variables.We used logarithmic transformation for the variables with skewed distributions such as TG and ACR to achieve a normal distribution. In order to analyze the baseline characteristics of study participants, we divided participants according to the presence/absence of MetS and compared the mean values of cardiometabolic risk factors using a Chi-squared test or t-test. To determine the optimal ACR cutoff points for metabolic syndrome and its components, we computed the receiver operating characteristic (ROC) curve. The optimal cutoff values were obtained from the maximal Youden’s index, calculated as (sensitivity + specificity—1) and the optimal combination of sensitivity and specificity. Multivariate logistic regression analyses were applied to examine the odds ratios (ORs) and 95% confidence intervals (CI) of MetS prevalence according to the ACR level. All statistical tests were 2-tailed and a p-value of <0.05 was considered to be statistically significant. Statistical analyses were performed using SAS software package version (9.2) for Windows (SAS Institute, Cary, NC, USA).

## Results

### Characteristics of participants

Table 1 shows the baseline characteristics of participants with and without MetS. Age, WC, SBP, DBP, FBG, and TG were higher in the MetS group than that in the non-MetS group (all p values < 0.001). ACR was higher in the MetS group than those in the non-MetS group (ACR: 8.1 ± 0.5 vs. 4.1 ± 0.1 mg/g, respectively; all p values< 0.001).

**Table 1:** General characteristics of subjects with and without MetS.

	MetS		
Variable	No	Yes	p value*
Unweighted (n)	6622	3028	
Age (years)	42.1 ± 0.3	54.4 ± 0.4	<0.001
Sex (male, %)	53.1 (0.7)	50.8 (1.1)	0.106
BMI (kg/m^2^)	23.0 ± 0.1	26.2 ± 0.1	<0.001
WC (cm)	78.6 ± 0.2	89.4 ± 0.2	<0.001
SBP (mmHg)	114.2 ± 0.3	128.3 ± 0.4	<0.001
DBP (mmHg)	74.7 ± 0.2	81.2 ± 0.3	<0.001
FBG (mg/dL)	92.0 ± 0.2	110.3 ± 0.7	<0.001
TC (mg/dL)	186.3 ± 0.6	198 ± 1.0	<0.001
TG** (mg/dL)	93.0 ± 1.5	180.5 ± 4.9	<0.001
HDL-C (mg/dL)	54.8 ± 0.2	45.1 ± 0.2	<0.001
LDL-C (mg/dL)	110.4 ± 0.5	113.7 ± 0.9	0.002
eGFR (mL/min/1.73 m^2^)	96.7 ± 0.3	88.6 ± 0.4	<0.001
ACR** (mg/g)	4.1 ± 0.1	8.1 ± 0.5	<0.001
Heavy drinker (yes, %)	9.8 (0.5)	13.0 (0.8)	<0.001
Current smoker (yes, %)	24.6 (0.8)	25.4 (1.1)	0.317
Regular exercise (yes, %)	20.0 (0.7)	16.0 (1)	0.001
Rural area (yes, %)	18.0 (2.0)	24.8 (2.6)	<0.001
Education level (yes, %)			
high school or more ≥10	79.0 (0.8)	52.3 (1.4)	<0.001
Monthly income (yes, %)			
lowest quartile	11.8 (0.6)	21.9 (1.1)	<0.001
DM medication (yes, %)	3.7 (0.3)	27.2 (1.1)	<0.001
HTN medication (yes, %)	14.0 (0.6)	60.5 (1.3)	<0.001

* p values were obtained using the Chi-squared test and Student’s t-test.

** Log transformation used to normalize data

Data are presented as mean ± SE or percentages (SE).

MetS, metabolic syndrome; BMI, body mass index; WC, waist circumference; SBP, systolic blood pressure; DBP, diastolic blood pressure; FBG, fasting blood glucose; TC, total cholesterol; TG, triglyceride; HDL-C, high-density lipoprotein-cholesterol; LDL-C, low-density lipoprotein-cholesterol; eGFR, estimated glomerular filtration rate; ACR, albumin-to-creatinine ratio

### ACR cutoff values according to the MetS components

ACR cutoff values analyzed by the ROC curve according to the number of MetS components and the individual MetS components (Table 2). The ACR cutoff value was 5.3 mg/g for subjects with ≥1 MetS component; 5.3 mg/g, for subjects with ≥2 components; 4.8 mg/g, for subjects with ≥3 components; 4.9 mg/g, for subjects with ≥4 components; and 5.8 mg/g, for subjects with all 5 MetS components. The ACR cutoff points for high BP, high BG, low HDL-C, high TG, and high WC were 5.3, 5.3, 5.2, 5.2, and 4.8 mg/g, respectively.

**Table 2:** The first ACR cutoff according to the number of and individual components of MetS.

Variables	Cutoff (mg/g)	Sensitivity (%)	Specificity (%)	AUC (95% CI)
Number of MetS components				
≥1	5.3	40.2	79.8	0.617 (0.608–0.627)
≥2	5.3	46.1	76.7	0.641 (0.632–0.651)
≥3	4.8	54.1	70.2	0.653 (0.644–0.663)
≥4	4.9	59.4	66.9	0.672 (0.663–0.682)
5	5.8	60.3	69.2	0.680 (0.671–0.689)
MetS components				
High WC	4.8	47.1	68.2	0.591 (0.581–0.600)
High BP	5.3	49.8	75.9	0.659 (0.650–0.669)
High BG	5.3	49.5	71.4	0.633 (0.624–0.643)
Low HDL-C	5.2	44.0	69.0	0.579 (0.569–0.589)
High TG	5.2	42.9	68.6	0.573 (0.563–0.583)

ACR, albumin-to-creatinine ratio; MetS, metabolic syndrome; AUC, area under the ROC curve; CI, confidential interval; WC, waist circumference; BP, blood pressure; BG, blood glucose; HDL-C, high-density lipoprotein-cholesterol; TG, triglyceride

### The mean ACR values and the percentage of subjects with ACR values over 5 mg/g

**Fig 1:**
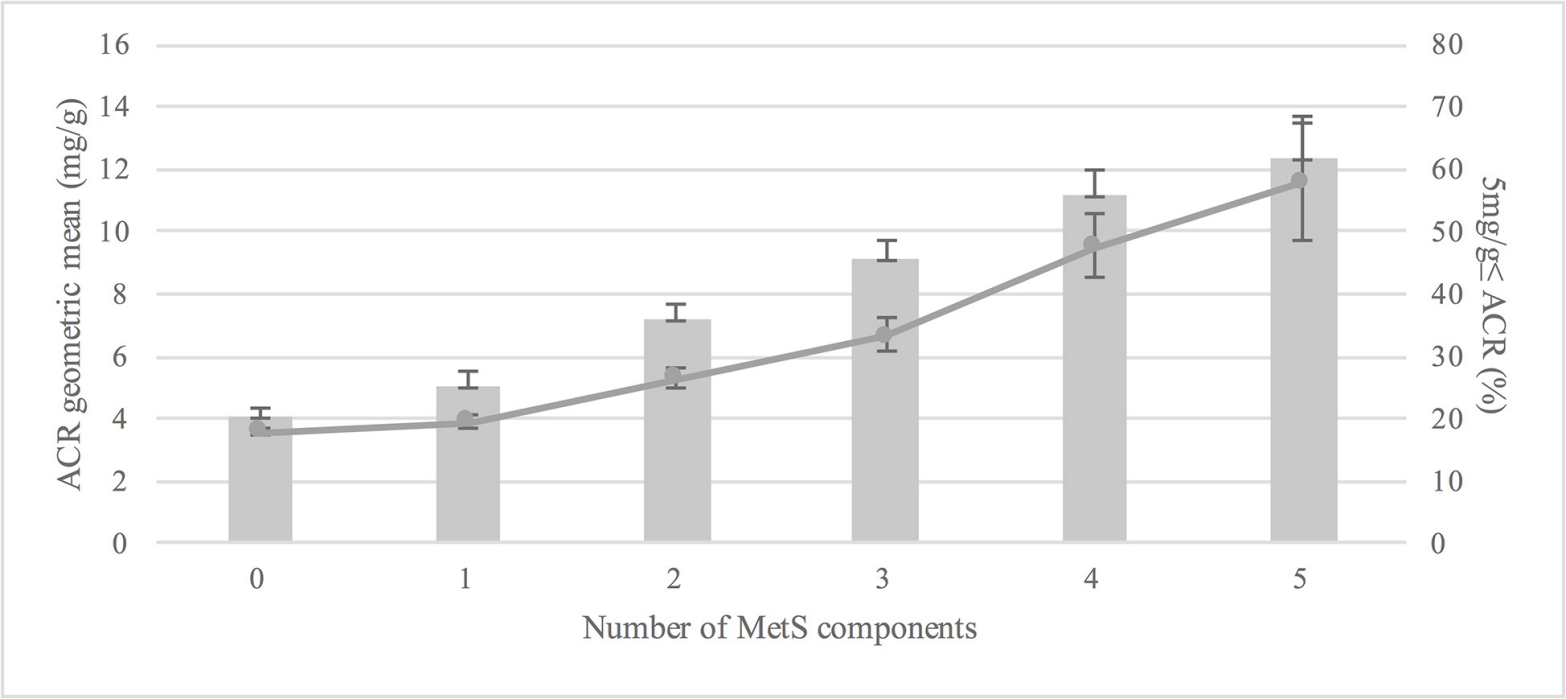
The geometric mean ACR values and the percentage of subjects with ACR values over 5 mg/g with an increase in the number of MetS components. All p for trends are <0.001. The geometric mean values of ACR were presented as a line, and the percentage of subjects with ACR values over 5 mg/g was presented as a bar graph. ACR, albumin-to-creatinine ratio; MetS, metabolic syndrome.

**Fig 2:**
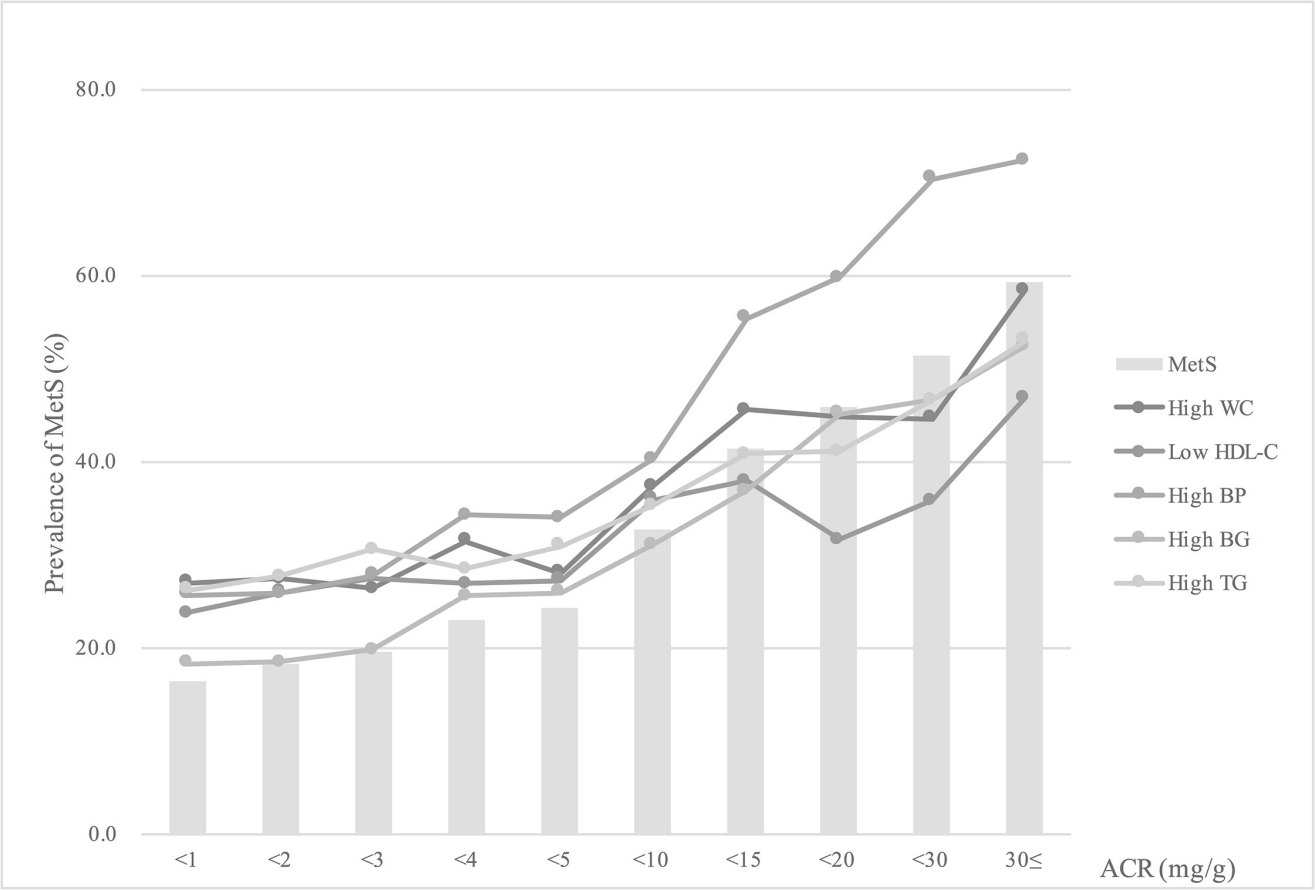
Graded association between ACR increase and the prevalence of MetS and its components. MetS, metabolic syndrome; ACR, albumin-to-creatinine ratio; WC, waist circumference; HDL-C, high-density lipoprotein-cholesterol; BP, blood pressure; BG, blood glucose; TG, triglyceride.

Geometric mean values of ACR increased according to the number of MetS components (presented as a line). Percentages of subjects whose ACR values were over 5 mg/g (presented as a bar graph) also increased according to the number of MetS components (p for trend < 0.001) (Fig 1). Fig 2 shows a graded association between ACR increase and the prevalence of MetS and its components. ACR <5mg/g did not show a significant increase in the prevalence of MetS or its components, but ACR levels ≥5 mg/g showed a remarkable increase.

### MetS prevalence according to the different ACR level

The adjusted ORs and 95% CIs for MetS prevalence were analyzed according to the different ACR level (Table 3). In model 1, which was adjusted for age and sex, the prevalence of MetS increased significantly even among people with very low ACR levels (≥1 mg/g). However, in model 4, which was adjusted for age, sex, BMI, smoking, alcohol consumption, exercise, and DM and HTN medication use, the prevalence of MetS significantly increased from the ACR levels of 4–5 mg/g (OR; 95% CI = 1.416; 1.041–1.926).

**Table 3:** Adjusted odds ratios of MetS prevalence according to the ACR level.

	ORs (95% CI) for MetS prevalence			
ACR (mg/g)	Model 1	Model 2	Model 3	Model 4
<1.0	1	1	1	1
1.0–1.9	1.303 (1.035–1.641)	1.146 (0.882–1.489)	1.129 (0.863–1.478)	1.066 (0.807–1.407)
2.0–2.9	1.415 (1.125–1.779)	1.419 (1.114–1.808)	1.382 (1.078–1.772)	1.271 (0.981–1.647)
3.0–3.9	1.557 (1.231–1.969)	1.410 (1.078–1.845)	1.388 (1.060–1.816)	1.318 (0.999–1.740)
4.0–4.9	1.707 (1.313–2.219)	1.610 (1.202–2.157)	1.583 (1.182–2.119)	1.416 (1.041–1.926)
5.0–9.9	2.140 (1.773–2.584)	1.935 (1.568–2.387)	1.893 (1.529–2.344)	1.722 (1.378–2.152)
10.0–14.9	2.831 (2.169–3.695)	2.423 (1.787–3.287)	2.408 (1.766–3.283)	2.146 (1.531–3.009)
15.0–19.9	3.386 (2.388–4.801)	2.665 (1.732–4.099)	2.359 (1.511–3.681)	1.930 (1.202–3.097)
20.0–29.9	3.850 (2.676–5.540)	3.464 (2.420–4.959)	3.225 (2.245–4.633)	2.635 (1.790–3.878)
≥30.0	4.742 (3.719–6.046)	3.370 (2.563–4.432)	3.349 (2.540–4.416)	2.428 (1.813–3.251)

Odds ratios and 95% confidence intervals were obtained by multivariable logistic regression analyses.

Model 1 was adjusted for age and sex.

Model 2 was adjusted for covariates of model 1 plus body mass index.

Model 3 was adjusted for covariates of model 2 plus smoking, alcohol intake, and regular exercise.

Model 4 was adjusted for covariates of model 3 plus use of medication for HTN and DM, and eGFR.

MetS, metabolic syndrome; ACR, albumin-to-creatinine ratio; OR, odd ratio; CI, confidential interval; eGFR, estimated glomerular filtration rate

### Comparison of the prevalence of MetS using different combination of MetS components

**Fig 3:**
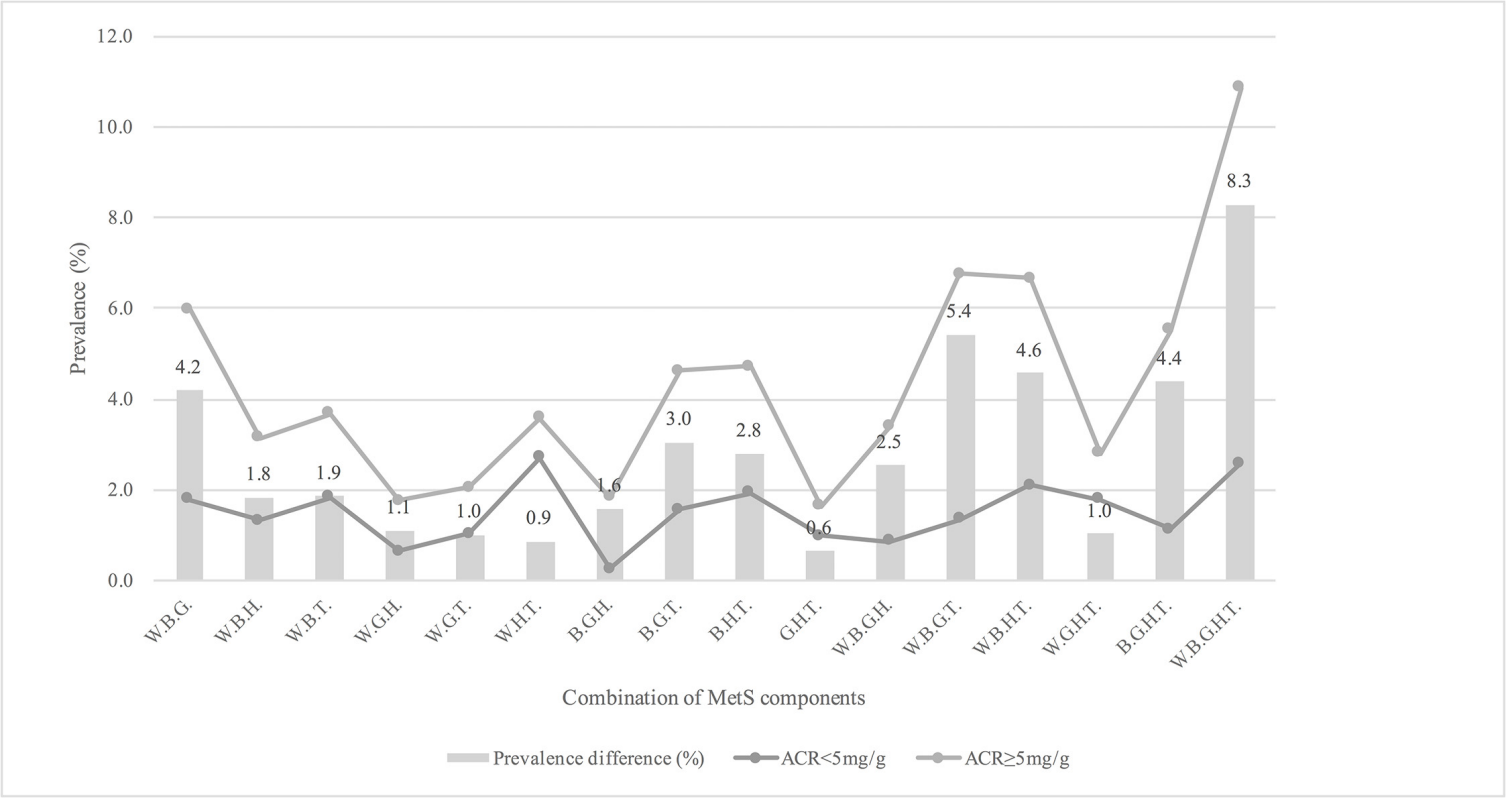
Comparison of the prevalence of MetS using different combination of MetS components with an ACR cutoff value of 5 mg/g. W, high WC; B, high BP; G, high BG; H, low HDL-C; T, high TG; MetS, metabolic syndrome; ACR, albumin-to-creatinine ratio.

We compared the prevalence of MetS using different combination of MetS components using an ACR cutoff value of 5 mg/g (Fig 3). Except for 2 combinations of high WC + low HDL-C + high TG and high BG + low HDL-C + high TG, a cutoff point of 5 mg/g associated with all other combinations (all p < 0.05). In subjects with ACR ≥5 mg/g, the most frequently reported pattern was a combination of high WC + high BP + high BG + low HDL-C + high TG. In subjects with ACR <5 mg/g the most frequently reported pattern was a combination of high WC + low HDL-C + high TG. Among combinations of 3 MetS components, the ACR level was most predictive of high WC + high BP + high BG and least predictive of high WC + high BG + high TG. Among combinations of 4 MetS components, the ACR level was most predictive of high WC + high BP + high BG + high TG and least predictive of high WC + high BG + low HDL-C + high TG (5.4% and 1.0%, respectively). The maximum difference predicted by the cutoff was in the combination of all MetS components (8.3%).

## Discussion

In this study, elevated ACR within the normal range was associated with a higher prevalence of MetS and its components. We identified a cutoff value for albuminuria of 4.8 mg/g, which significantly associated with the prevalence of MetS and its components in a representative sample of the South Korean population.

Many studies in different countries, including Korea, have shown an association between microalbuminuria and MetS [7–10]. Those studies use the standard definition of microalbuminuria. However, several recent studies suggested that individuals with high-normal albuminuria also have an increased risk of cardiometabolic risk factors including MetS [11–15]. Very low levels of microalbuminuria are also associated with coronary heart disease and cardiovascular mortality. The Third Copenhagen City Heart Study showed that microalbuminuria, defined as urinary albumin excretion >4.8 μg/min, was a strong and independent predictor of coronary heart disease and death in hypertensive patients and in participants with no history of coronary heart disease [19, 20]. Moreover, low levels of microalbuminuria (>5 μg/min) were associated with increased risk of MetS [4]. The Framingham Heart Study also reported that low-grade albuminuria (ACR ≥3.9 mg/g in men, ≥7.5 mg/g in women) was associated with a significant increase in the risk of the first cardiovascular event and mortality in non-hypertensive and non-diabetic subjects [21]. Ruggenenti et al. also found that even very low-grade albuminuria (ACR of 1–2 μg/min) was significantly associated with increased cardiovascular risk in type 2 DM patients [26]. In South Korea, Oh et al. reported that high-normal albuminuria (>4.87 μg/mg) was associated with significantly increased incidence of MetS in 4,338 middle-aged men participated in the medical check-up programs by themselves [14]. These findings show that the previous definition of microalbuminuria does not adequately reflect cardiometabolic abnormalities such as MetS

Some mechanisms may explain the association between normal range albuminuria and MetS and its components. One mechanism is an elevated vascular resistance. Vasodilatation in response to nitric oxide has been notably decreased at slightly elevated albuminuria in healthy subjects [27], and early carotid atherosclerotic lesions was independently associated with normal range albuminuria in type 2 DM patients [12]. Increased vascular renin-angiotensin system activity is also observed in the subjects with normal range albuminuria [28]. Another mechanism is based on the insulin resistance which also plays a role in the development of MetS. Insulin resistance could be involved in the development of microalbuminuria by increasing glomerular hydrostatic pressure, renal vascular permeability, renal sodium reabsorption, and aggregating glomerular hyperfiltration [29]. Endothelial dysfunction is also an important factor associated with hypertension and microalbuminuria [30].

There are some limitations to this study. First, this study is cross-sectional, making it difficult to explain causal relationships or describe clear mechanisms relating low-normal albuminuria to MetS and its components. Second, we used only one morning urine sample for evaluation of albuminuria. 24-h urine collection is generally recommended to assess albuminuria. However, a single morning urine sample is known to be correlated with 24-h urine albumin excretion rates [31].

Despite these limitations, this study has several strengths. First, it is a large epidemiologic study found the association between normal range albuminuria and MetS, based on the representative fraction of Korean population. Second, to the best of our knowledge, this is the first study to suggest a cutoff value of ACR associated with the prevalence of MetS and its components in the Korean population.

In conclusion, the prevalence of MetS and its components are increased in normal range albuminuria around 5 mg/g. Clinicians should carefully evaluate the risk of MetS in the patients with normal range albuminuria. Further prospective studies are needed to demonstrate a causal relationship between MetS and normal range albuminuria.
